# Sensor Technology for Sports Monitoring

**DOI:** 10.3390/s23020572

**Published:** 2023-01-04

**Authors:** Vesa Linnamo

**Affiliations:** Faculty of Sport and Health Sciences, University of Jyväskylä, 40014 Jyväskylä, Finland; vesa.linnamo@jyu.fi; Tel.: +358-40504-4800

Over the past decades, huge steps have been made in the development of sensor technology related to sports monitoring. Sensors are lighter, data transmission is mostly wireless, and software applications are more user-friendly. Wearable technologies have expanded from heart rate monitoring to, e.g., the use of inertial measurement units (IMUs) to detect motion and technique changes in different sports. IMUs combined with satellite systems also make speed detection and the analysis of an athlete's racing performance possible. New technologies have emerged for both laboratory and field conditions. In laboratory, it is easier to create more stable conditions, but if laboratory tests do not correspond to the actual sport performance on the field, one should be careful with the conclusions.

The challenge with any new sensor and/or algorithm is that it needs to be both valid and reliable. In other words, it needs to utilize applicable measures that are accurate and repeatable while additional measurements conditions remain unchanged. A common way to test new systems is to compare them against a “golden standard”, which is considered the most accurate available system for that purpose. If the measurement error is higher than the change expected to be seen in the performance variable, naturally, the system is not very useful. In a coaching situation, feedback to the athlete should, however, be given without too much delay and the coach needs to decide whether they are willing to compromise accuracy to save time.

This Special Issue “Sensor Technology for Sports Monitoring” addresses the topics raised above. It consists of ten papers related to all kinds of sensors that are currently being used for monitoring different sports. The topics vary from dancing to winter sports such as figure skating, alpine and cross-country skiing with para-athletic and able-bodied skiers, to summer sports such as football and kayaking.

The aim of the first article [1] in this Special Issue was to test the performance of an enhanced version of a prototype in a dynamic skiing-like bending simulation as well as in a proof-of-concept field measurement. It was concluded that combined with the high laboratory-based reliability and validity of the PyzoFlex^®^ prototype, it can be a potential candidate for smart ski equipment.

In [2], a system for detecting and visualizing the very important dance motions known as stops was introduced. Using a neural network to learn motion features, the system was able to detect stops and visualize them using a human-like 3D model with highly accurate stop detection results. Its effectiveness as an information and communication technology to support remote group dance practice was demonstrated.

Paper [3] is a pilot study introducing a motion analysis system to monitor alpine skiing activities during training sessions. Through five inertial measurement units (IMUs) placed on five points of the athletes, the angle of each joint was computed to evaluate the ski run. The aim of this work was to find a tool to support ski coaches during training sessions.

The aims of article [4] were to analyze the differences in physical demands of non-starter players regarding playing time during competition and to evaluate the physical demands of the compensatory training (MD + 1C) for substitute players in elite football. The study highlighted the importance of individualizing the workload of training sessions and suggested that the complementary session should be individualized according to the minutes played by the substitutes who are potentially under-loaded compared to starters.

In paper [5], the authors evaluated two approaches for estimating the total pro-pulsive force on a skier’s center of mass with double-poling and V2-skating skiing techniques using force measurement bindings, pole force sensors, and an eight-camera Vicon system for data collection. Both approaches properly estimated the trend of the force–time curve of the propulsive force, but due to its better accuracy, it was suggest-ed that an approach that calculates the forward component of the ground reaction force may be a more appropriate method than using the forward component of the translational force.

Article [6] presents the design and experimental evaluation of a non-invasive wearable sensor system that can be used to acquire crucial information about athletes’ performance during inline figure skating training. The system was able to detect when the jumps were performed and provided a live view of the data through a graphical user interface. The results confirmed its effectiveness in correctly detecting jumps, especially considering its compromise between precision and the overall cost of the equipment.

Papers [7,8] both studied kayaking. The aim of [7] was to investigate the magnitude and shape of the forces applied on the foot rest, foot strap, and paddle, and it was found that when comparing the best and worst kayakers’ performance, the best showed greater magnitudes of force and greater synchronization of the peak forces. It was recommended that analyses of the force–time curves, including not only the forces applied by the kayaker on the paddle but also the ones applied on the foot rest and strap, should be considered relevant in terms of technique analyses. In article [8], the aim was to compare key variables of paddle strokes measured by a commercial Trainesense SmartPaddle^®^ against the strain-gauge shaft and investigate how these variables are associated with the velocity of the boat among national-level canoe polo players. It was concluded that the SmartPaddle can provide promising information on key stroke variables when compared to the strain-gauge paddle shaft. In the future, this could be an interesting tool for biomechanical research and daily kayaking coaching in real open water conditions. 

The topic of article [9] was related to para-athletic cross-country (XC) skiing. The paper's aims were to evaluate the feasibility of a framework based on micro-sensor technology for, firstly, in-field analyses of performance and, secondly, sub-technique selection in the field by using it to compare different parameters between elite standing para-athletic and able-bodied XC skiers during a classical skiing race. The data from a global navigation satellite system and inertial measurement unit were integrated to compare time loss and selected sub-techniques as a function of speed. It was found that there can be different speed thresholds in the classical sub-techniques for para-athletic skiers versus able-bodied skiers. It was suggested that the framework could provide a point of departure for large-scale international investigations of performance and related factors in para-athletic XC skiing.

The aim of the final paper [10] of this Special Issue was to develop a novel ski demonstrator and to conceptualize and validate an empirical curvature model. PyzoFlex^®^ technology-based sensor foils were attached to the upper surface of an al-pine ski, and a self-developed instrument was used as a data acquisition device. Although the new measuring system offered good accuracy and high precision, it was suggested that a transfer into the field is only allowed to a limited extent since the scope of the curvature model has not yet been definitely determined. The authors concluded that high laboratory-related reliability and validity of their novel ski prototype featuring PyzoFlex^®^ technology make it a potential candidate for on-snow application, such as smart skiing equipment.

In order to be able to analyze and give proper advice on sport techniques, it is important to understand the biomechanical and physiological demands of different sports. Thus, various sport monitoring systems are needed to help athletes achieve optimal performance. As this Special Issue shows, several developments in this field have been made all over the world in different research institutes. The work, however, continues and should continue to make sensors in this field even more accurate and user-friendly in the future.
